# Sex Effects on Smoking Cue Perception in Non-Smokers, Smokers, and Ex-Smokers: A Pilot Study

**DOI:** 10.3389/fpsyt.2016.00187

**Published:** 2016-11-17

**Authors:** Davide Zanchi, Arthur Brody, Stefan Borgwardt, Sven Haller

**Affiliations:** ^1^Department of Psychiatry (UPK), University of Basel, Basel, Switzerland; ^2^Department of Psychiatry, University of California at Los Angeles, Los Angeles, CA, USA; ^3^Department of Research, VA Greater Los Angeles Healthcare System, Los Angeles, CA, USA; ^4^Affidea Centre de Diagnostic Radiologique de Carouge CDRC, Geneva, Switzerland; ^5^Faculty of Medicine, University of Geneva, Geneva, Switzerland; ^6^Department of Surgical Sciences, Radiology, Uppsala University, Uppsala, Sweden; ^7^Department of Neuroradiology, University Hospital Freiburg, Freiburg, Germany

**Keywords:** nicotine, tobacco, fMRI, sex, females, males

## Abstract

**Introduction:**

Recent neuroimaging research suggests sex-related brain differences in smoking addiction. In the present pilot study, we assessed gender-related differences in brain activation in response to cigarette-related video cues, investigating non-smokers, smokers, and ex-smokers.

**Methods:**

First, we compared 29 females (28.6 ± 5.3) vs. 23 males (31.5 ± 6.4), regardless of current smoking status to assess global gender-related effects. Second, we performed a *post hoc* analysis of non-smokers (9 females and 8 males), smokers (10 females and 8 males), and ex-smokers (10 females and 7 males). Participants performed a block-design functional magnetic resonance imaging paradigm contrasting smoking with control cue video exposures. Data analyses included task-related general linear model, voxel-based morphometry of gray matter (GM), and tract-based spatial statistics of white matter (WM).

**Results:**

First, the global effect regardless of current smoking status revealed higher activation in the bilateral superior frontal gyrus and anterior cingulate cortex (ACC) for females compared to males. Second, the analysis according to current smoking status demonstrated higher activation in female vs. male smokers vs. non-smokers in the superior frontal gyrus, anterior and posterior cingulate cortex, and precuneus, and higher activation in female vs. male ex-smokers vs. non-smokers in the right precentral gyrus, in the right insula and ACC. No structural differences were found in GM or WM.

**Conclusion:**

The current study identifies gender-related brain functional differences in smokers and ex-smokers compared to non-smokers. The current work can be considered as a starting point for future investigations into gender differences in brain responses to cigarette-related cues.

## Introduction

On the basis of recent data from the WHO, it has been estimated that 250 million women worldwide smoke daily (1). Between 1950 and 2000, approximately 10 million women died due to smoking (2), and it has been estimated that, between 2002 and 2030, this figure will have increased to 40 million (3). As several works on addiction demonstrate, sex plays an important role in addictive behaviors (4). In fact, women progress more rapidly to dependence and suffer more frequent co-occurring depression and anxiety than men (5–7).

Furthermore, neuroimaging techniques, such as positron emission tomography (PET) and functional magnetic resonance imaging (fMRI), allowed the detection of gender-related differences in brain functions associated with addiction, identifying in dependent women higher activation in frontal regions related to craving stimuli compared to men (8, 9).

Recent fMRI studies focusing on heavy smokers established a link between blood oxygenated level-dependent (BOLD) signal in brain areas related to nicotine craving and the sex of participants (10, 11). In particular, women showed higher activation in the striatum and in the superior frontal gyrus, while men had higher activation in the hippocampus and orbitofrontal cortex. Furthermore, sex-related differences were suggested also in cerebral blood flow (CBF) during exposure to smoking-related cues (12).

Although gender effects during nicotine addiction are suggested, none of the previous studies contrasted male and female smokers to a non-smoking group to control for the effects of stimulus administration. Moreover, the previous works investigated current smokers, while sex-related differences during long-term nicotine abstinence (in ex-smokers) have yet to be investigated.

Given these previous findings, we hypothesized the presence of gender-related brain functional differences during tobacco dependence and long-term tobacco abstinence. fMRI in a task-based paradigm, contrasting smoking with control cue videos was used in three populations: non-smokers, active smokers, and ex-smokers (more than 1 year of smoking abstinence). In a first step, we analyzed global gender-related differences in smoking cue processing regardless of the current smoking status by contrasting 29 females vs. 23 males. In fact, as researches on passive smoking demonstrate, it is not an unusual situation that even non-smokers are exposed to smoking cues on a regular basis (13). In the second step, we further analyzed gender-related differences depending on the current smoking status of smokers and ex-smokers compared to non-smokers.

## Materials and Methods

The presented work is an extension of an fMRI dataset previously published by Zanchi et al. (14). Details of the study population, fMRI acquisition protocol, task, and fMRI group analyses and results are defined in the abovementioned publication. Details specific to the current study will be described here.

### Participants

This study was approved by the local ethics committee (University Hospital of Geneva). All the experimental procedures were carried out in accordance with the approved guidelines and with the principles of the Declaration of Helsinki. All the participants gave written informed consent prior to inclusion. Fifty-one subjects were recruited by word of mouth and local advertising. The inclusion criterion for non-smoking status was the absence of any regular lifetime use of tobacco. Inclusion criteria for smokers were: (a) 10 or more cigarettes smoked per day over at least 2 years, (b) no intention to quit smoking in the next month, and (c) ability to maintain 15 min of smoking abstinence prior to scanning. Inclusion criteria for male and female ex-smokers were: (a) abstinence from cigarettes for more than 12 months prior to the study and (b) previous smoking period of at least 2 years with 10 or more cigarettes per day. Non-smokers, smokers, and ex-smokers groups matched for gender and age, with smokers and ex-smokers matching also for years of smoking and number of cigarettes smoked per day. Within each group, males and females were also matched for age, years of smoking, and years of abstinence. Exclusion criteria for all participants were history of drug or excessive alcohol abuse/dependence, major medical disorders, and use of medications at the time of brain scanning that could affect brain function (e.g., psychotropics, stimulants, or β-blockers). The final sample included 9 female (mean age 27.19, SD ± 4.2) and 8 male (mean age 33.41, SD ± 4.2) non-smokers, 10 female (mean age 28.31, SD ± 5.7) and 8 male smokers (mean age 29.84, SD ± 5.2), and 10 female (mean age 30.93, SD ± 6.3) and 7 male (mean age 29.58, SD ± 4.6) ex-smokers (Table 1).

**Table 1:** **Essential demographic parameters (mean and SD) of the included study**.

Demographic variables and related statistics												
	Females	Males	Significance	Female non-smokers	Male non-smokers	Significance	Female smokers	Male smokers	Significance	Female ex-smokers	Male ex-smokers	Significance
Cases	29	23	–	9	8	–	10	8	–	10	7	–
Age	28.87 ± 0.99	31.87 ± 1.56	*P* = 0.1	27.19 ± 4.2	33.41 ± 4.2	*P* = 0.06	28.31 ± 5.7	29.84 ± 5.2	*P* = 0.29	30.93 ± 6.3	29.58 ± 4.6	*P* = 0.67
Years of smoking	9.06 ± 1.1	12.83 ± 2.35	*P* = 0.11	–	–	–	10.43 ± 4.4	10.3 ± 5.5	*P* = 0.28	8 ± 4.3	9.8 ± 5.1	*P* = 0.52
Cigarettes a day	12.22 ± 0.95	14.11 ± 1.32	*P* = 0.26	–	–	–	11.89 ± 2	10.7 ± 5.2	*P* = 0.34	12.56 ± 5.5	11.75 ± 2.4	*P* = 0.78
Years of abstinence	4.20 ± 1.04	2.50 ± 0.86	*P* = 0.35	–	–	–	–	–	–	4.2 ± 3.2	2.5 ± 1.7	*P* = 0.35
Rating scores	33.84 ± 4.89	32.18 ± 7.67	*P* = 0.85	11.12 ± 15.7	9.862 ± 13	*P* = 0.85	60.4 ± 17.2	74 ± 10.1	*P* = 0.17	30 ± 19.8	35 ± 23.2	*P* = 0.69

### Functional Magnetic Resonance Imaging Acquisition and Paradigm

Images were obtained using a 3-T scanner (Trio; Siemens, Erlangen, Germany) with a standard 32 channel head coil. Subjects underwent an fMRI examination including a functional scan, a 3D T1-weighted structural scan, and a diffusion tensor imaging (DTI) sequence. For technical details regarding the fMRI parameters, please see Ref. (14). During the functional run, an on–off block-design with two active conditions (smoking and control cue videos) and a neutral condition (cross displayed) developed by Brody et al. (15) were shown as in the previous work (14). After each video, a visual analog scale was presented in which the subjects rated the craving perceived during the video cues (0 = no craving, 100 = maximum craving).

### Statistical Analysis

Statistical analyses were conducted using GraphPad Prism (Version 6, GraphPad Software, San Diego, CA, USA) and FSL (Version 5.0.6, FMRIB, Oxford, UK).

### Analysis of Demographic and Behavioral Data

First, a D’Agostino–Pearson omnibus test was performed to check for normal distribution. Then, we investigated demographic and behavioral differences between males and females overall separately and for each group (non-smokers, smokers, and ex-smokers). Normally distributed variables, notably age and years of smoking, were analyzed using unpaired *t*-tests. Non-normally distributed variables, notably number of cigarettes a day and rating scores for the smoking cue videos, were tested using Mann–Whitney non-parametric tests.

### Task-Related General Linear Model

Processing and analysis of imaging data were performed using FSL FEAT (fMRI Expert Analysis Tool version 6.00^1^). Preprocessing included brain extraction using FSL’s brain extraction tool (BET) (16), motion correction using FSL’s intra-modal motion correction tool (MCFLIRT) (17), and smoothing using FSL’s noise reduction uses non-linear filtering (SUSAN) (18). A general linear model (GLM) was employed at four levels of analyses. At the first level, the contrast of “smoking vs. control videos” was calculated individually for each run of each participant using a fixed-effects analysis. Then, at the second level, a fixed-effects analysis combined both runs of each participant. At the third level, a random-effects analysis was performed to investigate group differences between males and females first regardless of the smoking status and then for each group separately: non-smokers, smokers, and ex-smokers. Finally, at the fourth level, a random-effects analysis was performed to investigate gender-related group differences between non-smokers, smokers, and ex-smokers. The results were corrected for multiple comparisons by threshold-free cluster enhancement (19). *P* values <0.05 were considered as significant.

Moreover, Pearson’s correlations were performed between imaging results and craving scores related to smoking videos-cues for each group. *P* values <0.05 were considered as significant.

### VBM and TBSS Analyses

To assess gray matter (GM) and white matter (WM) gender-related differences, a voxel-based morphometry (VBM) and a tract-based spatial statistic (TBSS) analysis were performed in FSL (FSL Version 5.0.6^2^) using standard processing steps (20). At group level, a voxel-wise GLM was implemented using permutation-based non-parametric testing (Randomize, part of FSL). Results were corrected for multiple comparisons using TFCE (19), and *P* values <0.05 were considered as significant.

## Results

### Demographics and Rating Scores

No significant differences were found in the demographic variables (age, years of smoking, cigarettes a day, and years of abstinence) and in the rating scale scores of smoking videos between males and females for each group (Table 1).

### Task-Related General Linear Model

In the task-related GLM, we considered the contrast of smoking vs. control cue videos.

**Figure 1:**
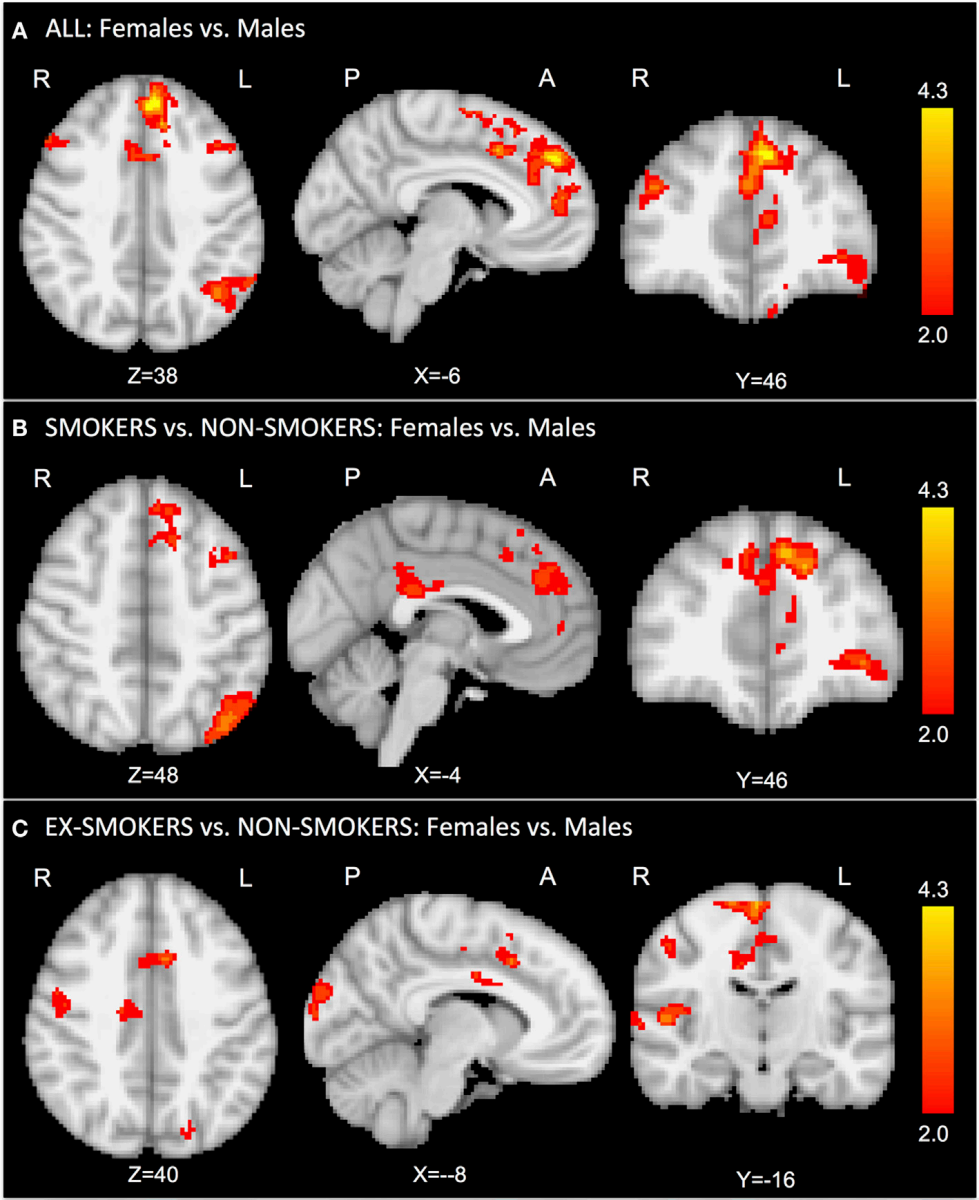
**In the task-related GLM, we considered the contrast of smoking vs. control cue videos**. The comparison “females vs. males” revealed significantly greater activation in the bilateral superior frontal gyrus and anterior cingulate cortex for females **(A)**. The comparison “female vs. male smokers vs. non-smokers” revealed significantly greater activation in the superior frontal gyrus, anterior cingulate cortex, and precuneus for female smokers **(B)**. The comparison “female vs. male ex-smokers vs. non-smokers” revealed increased activation in the right insula and anterior cingulate cortex for female ex-smokers **(C)**. No significant results were found for the contrast “female vs. male non-smokers.”

First, we assessed the main effect of gender regardless of current smoking status. The comparison “females vs. males” revealed significantly greater activation in the bilateral superior frontal gyrus and anterior cingulate cortex (ACC) for female smokers (Figure 1A; Table S2A in Supplementary Material).

Second, we performed a *post hoc* analysis of current smoking status.

The comparison “female vs. male smokers vs. non-smokers” revealed significantly greater activation in the superior frontal gyrus, ACC, and precuneus for female smokers (Figure 1B; Table S2B in Supplementary Material). The comparison “female vs. male ex-smokers vs. non-smokers” revealed increased activation in the right precentral gyrus, in the right insula, and ACC for female ex-smokers (Figure 1C; Table S2C in Supplementary Material). No significant results were found in the non-smokers group for the contrast “females vs. males.”

Moreover, no significant correlations were found between imaging results and craving scores for smoking videos for each group.

### VBM and TBSS Analysis

Voxel-based morphometry analysis of GM density and TBSS analysis of WM revealed no statistical differences between males and females for non-smokers, smokers, and ex-smokers.

## Discussion

The present exploratory study investigates the presence of gender-related functional brain differences in smokers and ex-smokers compared to non-smokers, underpinning smoking cue responses. Results reveal that females compared to males show significantly higher activations in brain areas related to cigarette craving.

In the first step, we assessed gender-related differences in smoking cue processing regardless of current smoking status. In fact, as researches on passive smoking demonstrate, it is not an unusual situation that even non-smokers are exposed to smoking on a regular basis (13). This can determine brain responses to smoking cues. Female smokers and ex-smokers show higher activation in the bilateral superior frontal gyrus and ACC. Our results are consistent with previous studies, demonstrating the involvement of these brain regions in cue-induced cigarette craving (15, 21).

In the second step, we performed sub-group analyses according to the current smoking status.

To investigate more specifically gender-related differences in an addicted population, we contrasted the smokers group with non-smokers. GLM results show a persistent higher activation of the superior frontal gyrus, ACC, and precuneus in female vs. male smokers when compared to female vs. male non-smokers. These results indicate gender-related differences in brain regions related to smoking cue perception in the smoker group. Our findings are consistent with previous studies of cocaine addiction, in which anterior and posterior cingulate cortex and more in general frontal cortical areas are demonstrated to be more active in cocaine addicted women compared to men (8, 9). Studies on gambling addiction also confirm this trend, revealing higher activation in frontal areas in females than in males (22).

Moreover, focusing on nicotine addiction, our results confirm a previous study of McClernon (11) that investigated gender effects in smokers. Frontal areas, as the superior frontal gyrus, are more activated in female smokers compared to male smokers during smoking-related stimuli. Additionally, in line with this previous work, no significant differences were found in craving scores between females and males. Therefore, we agree with the interpretation given by McClernon, suggesting that women and men engage different neural circuits in the processing of craving cues that lead ultimately to similar levels of subjective craving.

However, it is interesting to note that, in contrast with a study of Wetherill and colleagues (12), no significant differences at the brain level were identified in male vs. female smokers. This can be explained by the presence in our study of a control group that allows us to control for non-specific effects of cue exposure found in non-smokers. Moreover, BOLD signal and CBF are different measures of brain activity, and their results cannot be directly comparable (23).

We also found differences between females and males in the ex-smokers when contrasted to non-smokers. In line with the abovementioned tendency, women show greater activation than men in cortical frontal regions related to craving, such as the ACC and insula. Our results extend previous findings (11), suggesting gender differences in brain functions as present also during long-term nicotine abstinence. However, only two studies have been conducted on ex-smokers (14, 24); therefore, the present work represents a starting point for further investigations.

Interestingly, no significant GM and WM gender-related differences were found in the three groups. Subtle gender-related alterations in GM and WM were documented in studies with substantially larger sample size (25). The absence of significant differences in GM or WM in our study indicates that structural abnormalities are no substantial confounding factor in our sample.

Finally, at the clinical level, further research is necessary to investigate whether gender differences in neural responses to smoking cues perception predict clinical outcomes. Treatments that amend neural responses to smoking cues may be effective adjunctive therapies for smokers. Furthermore, smoking cue perception in frontal and cingulate brain regions may provide a novel target approach for development of new cessation treatments of smokers.

The modest number of subjects per sub-group is the main limitation of the present work that is intended to be a pilot study. However, this aspect of the study is partially compensated for by matching the demographic variables within and across the three groups. The small sample size can explain also the absence of significant correlations between imaging results and craving scores. However, it is interesting to notice that craving scores for smokers were the highest compared to ex-smokers (intermediate position) and non-smokers (lowest level). These results show that smokers indeed perceived more craving than non-smokers and ex-smokers during smoking cue videos. This finding was discussed already in our previous article (14), and it is in line with previous studies using the same cue videos (15). The absence of a direct correlation in the present work can be explained by the already acknowledge small sample size that decreases the statistical power. Another explanation could be the dependence between craving and group discrimination. In fact the results of the analyses of craving are confounded by group attribution (smokers already have higher craving). An option to investigate this would be to perform an orthogonalization during the analyses, but the problem of interpretation of the results would rise, in particular with such a low number of subjects. We recommend this for future works using a bigger sample size Furthermore, other factors, such as genetics, hormonal status of females, other drug use, etc., could influence findings and were not assessed in this study. It is also important to notice that even though differences in age between male and female non-smokers are not present, the significance level is approached. However, no results were found comparing female and male non-smokers both in the craving scores and at behavioral level. Finally, significant results were found with a small number of participants, suggesting the promising nature of this research.

## Conclusion

The current study identifies for the first time gender-related brain functional differences in smokers and ex-smokers when compared to non-smokers. The current work can be considered as a starting point for future investigations on this topic.

## Ethical Approval

This study was approved by the Ethics Committee of the University Hospital of Geneva. All the experimental procedures were carried out in accordance with the approved guidelines and with the principles of the Declaration of Helsinki. All the participants gave written informed consent prior to inclusion.

## Availability of Data and Materials

The dataset supporting the conclusions of this article is available at the University Hospital of Geneva. For repository, please contact Prof. Sven Haller.

## Author Contributions

Conceived and designed the experiments: DZ and SH. Performed the experiments: DZ and SH. Data analyses: DZ, AB, and SH. Writing the paper: DZ, AB, SB, and SH. All the authors read and approved the final manuscript.

## Conflict of Interest Statement

The authors declare that the research was conducted in the absence of any commercial or financial relationships that could be construed as a potential conflict of interest.

## Footnotes

^1^http://fsl.fmrib.ox.ac.uk/fsl/fslwiki/FEAT.

^2^http://fsl.fmrib.ox.ac.uk.

## Abbreviations

ACC, anterior cingulate cortex; BET, brain extraction tool; BOLD, blood oxygenated level dependent; CBF, cerebral blood flow; FEAT, FMRI Expert Analysis Tool; fMRI, functional magnetic resonance imaging; FSL, FMRIB software library; GLM, general linear model; GM, gray matter; MCFLIRT, intra-modal motion correction tool; MRI, magnetic resonance imaging; PET, positron emission tomography; SUSAN, noise reduction uses non-linear filtering; TBSS, tract-based spatial statistics; VBM, voxel-based morphometry.
